# ClinFly: an all-in-one method to translate, de-identify, and summarize medical reports in HPO format

**DOI:** 10.1093/nargab/lqaf145

**Published:** 2025-11-03

**Authors:** Lucas W Gauthier, Marjolaine Willems, Nicolas Chatron, Camille Cenni, Pierre Meyer, Valentin Ruault, Constance Wells, Enody Gernet, Caroline Dunoyer, Quentin Sabbagh, Claire Bardel, David Genevieve, Kevin Yauy

**Affiliations:** Genetics Department, AURAGEN, Hospices Civils de Lyon, 69500 Bron, France; Inserm U1183, IRMB, Reference center for congenital anomalies, Clinical Genetic Unit, CHU Montpellier, Univ Montpellier, 34000 Montpellier, France; Inserm U1183, IRMB, Reference center for congenital anomalies, Clinical Genetic Unit, CHU Montpellier, Univ Montpellier, 34000 Montpellier, France; Genetics Department, AURAGEN, Hospices Civils de Lyon, 69500 Bron, France; Institute NeuroMyoGène, Laboratoire Physiopathologie et Génétique du Neurone et du Muscle, CNRS UMR 5261–INSERM U1315, Université Claude Bernard Lyon 1, 69000 Lyon, France; Clinical Cytology and Genetics Department, CHU Nîmes Carémeau, Univ Montpellier, 30000 Nîmes, France; Department of Pediatric Neurology, CHU Montpellier, PhyMedExp, CNRS, INSERM, Univ Montpellier, 34000 Montpellier, France; Inserm U1183, IRMB, Reference center for congenital anomalies, Clinical Genetic Unit, CHU Montpellier, Univ Montpellier, 34000 Montpellier, France; Inserm U1183, IRMB, Reference center for congenital anomalies, Clinical Genetic Unit, CHU Montpellier, Univ Montpellier, 34000 Montpellier, France; Medical Information Department, CHU Montpellier, 34000 Montpellier, France; Medical Information Department, CHU Montpellier, 34000 Montpellier, France; Inserm U1183, IRMB, Reference center for congenital anomalies, Clinical Genetic Unit, CHU Montpellier, Univ Montpellier, 34000 Montpellier, France; NGS-HCL Platform, Bioinformatics Unit, Hospices Civils de Lyon, 69500 Bron, France; Inserm U1183, IRMB, Reference center for congenital anomalies, Clinical Genetic Unit, CHU Montpellier, Univ Montpellier, 34000 Montpellier, France; Clinical Genetic Unit, ERIOS, CHU Montpellier, 34000 Montpellier, France; MAB, LIRMM, Univ Montpellier, 34000 Montpellier,France

## Abstract

Genomic medicine relies on precise phenotyping and global data sharing, particularly in the context of rare diseases. However, exchanging medical reports across language barriers remains a major challenge. Manual annotation with the Human Phenotype Ontology (HPO) is also prone to inconsistency and incompleteness, risking the loss of clinically relevant information and potential misdiagnoses. We present ClinFly, an open-source pipeline that automatically de-identifies, translates, and summarizes medical reports into HPO terms, in compliance with health-data privacy standards and FAIR principles. In a multicenter, prospective evaluation, we benchmarked ClinFly against physician annotation for de-identification and phenotypic summarization of 50 non-English medical reports. The method achieved a recall of 99% and precision of 77% for protected health information (PHI) de-identification. For HPO summarization, high-confidence predictions reached a recall of 49% and precision of 92%, improving to 78% recall when including low-confidence terms, with an average of 6.6 HPO terms captured per report. ClinFly enables efficient, automated de-identification of PHI and summarization in HPO format to support genomic medicine across language barriers.

## Graphical Abstract

**Graphical Abstract:**
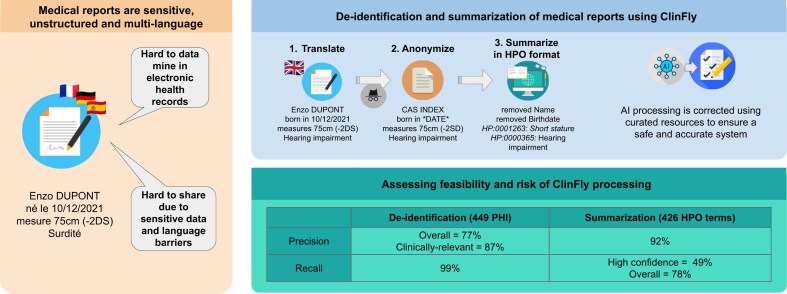

## Introduction

Precision or genomic medicine requires precise phenotyping and structured electronic health records based on clinical data sharing, especially in the context of rare diseases, for which matching patients worldwide is crucial [1]. In particular, medical reports contain critical information about a patient’s condition, so they are crucial for communication between healthcare providers [2]. However, sharing reports between providers who speak different languages can be challenging and time-consuming, especially if the reports need to be translated and de-identified to protect patient privacy [3]. Moreover, medical reports are unstructured text, which is difficult to exploit in precision medicine.

As an alternative, the worldwide community in genomic medicine adopted the Human Phenotype Ontology (HPO), whereby physicians can use a common language with machines to describe the patient’s symptoms [4]; other existing ontologies are disease-based and not symptom-based [5]. Moreover, HPO has gathered a large symptom-gene-association knowledge of genetic diseases, and it supports multiple languages, including English, Chinese, and Spanish. Sharing HPO terms summarizing clinical descriptions has been found effective in discovering new diseases via MatchMaker Exchange [6] by matching the similarity of symptoms between patients and is a key element to increase diagnostic yield by using computational phenotype analysis for genome sequencing analysis [7]. However, when evaluated, HPO terms provided by physicians considerably vary, which highlights the need for standardized practices and reproducibility to enhance communication efficiency. Additionally, these terms are often incompletely noted in records, which results in loss of clinical information [8].

Recent advancements in artificial intelligence (AI), particularly in natural language processing, offer promising capabilities for accurately translating and securely sharing medical information through effective de-identification [9–11]. While major cloud providers offer comprehensive protections and certifications ensuring compliance with GDPR, HIPAA, and other regulations, concerns around the sovereignty and control of healthcare data and computers persist, particularly in regions with stringent data localization requirements. Therefore, recommendations are to keep protected health information (PHI) in-house and processing of medical data offline [12]. Achieving on-premise translation, de-identification, and structuring of clinical data is essential to efficiently enable information sharing between collaborating physicians across institutions worldwide, leverage clinical data warehouses, and apply algorithms to uncover and better understand diseases. Multiple tools have been developed to individually translate, de-identify, and summarize medical reports into HPO terms [13, 14]. However, to our knowledge, no easily deployable tool integrates all of these functionalities.

Here we introduce ClinFly, an open-source pipeline specifically designed for the clinical genetics workflow to translate, de-identify, and summarize medical reports using HPO terms in line with FAIR principles (findability, accessibility, interoperability, and reuse of digital assets) while ensuring data privacy and security [15]. We assessed this method by comparison with a physician’s performance with 50 medical reports from clinical geneticists.

## Materials and methods

### Overview of ClinFly function

**Figure 1.**
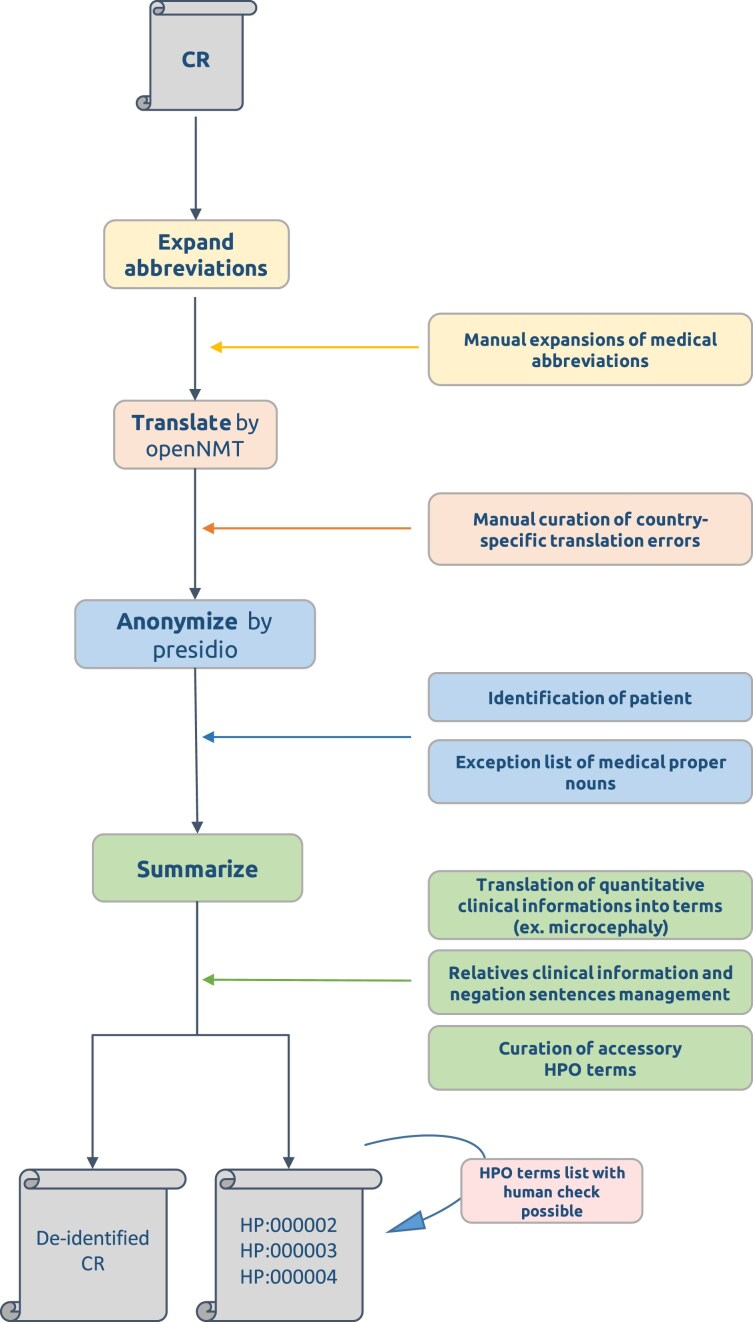
ClinFly pipeline. ClinFly can translate, de-identify, and summarize clinical reports using Human Phenotype Ontology (HPO) terms, thus ensuring compliance with medical data privacy standards. The output includes a de-identified translated clinical report and a summary report in HPO format.

**Figure 2.**
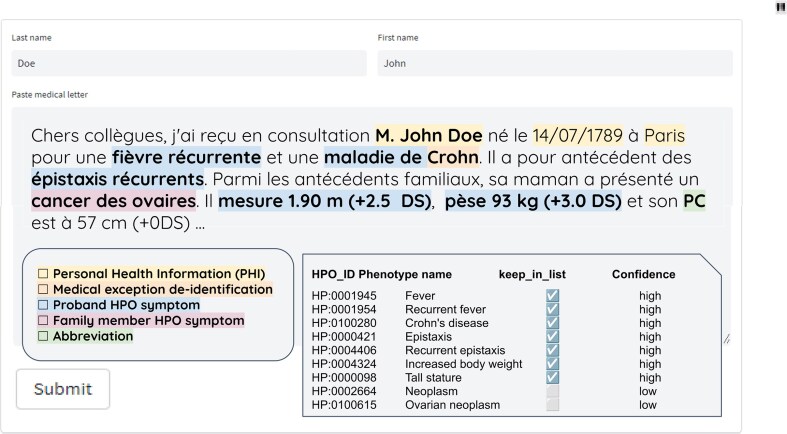
Framework example. Example of a French medical report, including personal health information in yellow, the proper medical name to exclude from de-identification in orange, the proband Human Phenotype Ontology (HPO) symptom in blue, the family member HPO symptom in pink, and the abbreviation in green, summarized in HPO terms with confidence level flags.

ClinFly enables clinicians locally to first, translate medical reports; second, perform de-identification; and finally, extract HPO terms from the processed reports. Reports were processed according to the following pipeline: (i) Abbreviations were first automatically expanded based on a manually curated dictionary with abbreviations, detailed in Supplementary Methods S1 and Supplementary Table S1; (ii) expanded text was translated into English by using the Marian translator, an open-source neural machine translation tool developed by OpenNMT [16] (https://marian-nmt.github.io); (iii) translated and corrected text was de-identified from PHI using Microsoft’s Presidio (https://microsoft.github.io/presidio/); and (iv) translated and de-identified text was summarized in HPO format, including quantitative data for symptoms, by using an upgraded version of ClinPhen [17] and flagged “high-confidence” or “low-confidence” according to the possibility of being associated with a relative-concerned or negative sentence (Fig. 1). ClinPhen manages synonym identification and lemmatization for HPO extraction. An example of the method processing of a typical French medical report is in Fig. 2. Additional details available are in Supplementary Methods S2.

### Hybrid processing with human correction of medical terms and databases

To avoid losing clinical information, we implemented in ClinFly a hybrid process involving both human-based review and AI techniques, using French as a proof of concept. To identify and prevent de-identification errors, we compiled a list of 2 998 medical-associated proper names, 4939 official drugs, and 109 804 gene symbols from various databases, including the Online Catalog of Human Genes and Genetic Disorders (OMIM) phenotype titles (March 2023 update, https://omim.org/), HPO terms (March 2023 release, https://omim.org/statistics/update), Agence nationale de sécurité du médicament et des produits de santé lists of official medication in allopathy without homeopathy and herbal medicine (March 2023 update, https://ansm.sante.fr/uploads/2024/01/16/20240116-medicaments-acces-direct-annexe-1-16-01-2024.pdf), and the complete HUGO Gene Nomenclature Committee-approved gene dataset (March 2023 update, https://ftp.ebi.ac.uk/pub/databases/genenames/hgnc/tsv/hgnc_complete_set.txt) to exclude them from de-identification. Given that the method location detection was insufficient concerning French territories, we used a list of 34 981 proper names for French cities, departments, and regions from the French National Institute of Statistics and Economic Studies official geographic code (https://www.insee.fr/fr/information/6051727, January 2022 release) to force de-identification. All details are in Supplementary Methods S1.

### Assessment on real-world medical reports

We conducted a multicenter prospective study at the University Hospital Centers of Montpellier and Nîmes (France) to evaluate ClinFly’s performance in generating English translations, performing de-identification, and extracting symptom summaries in HPO format from non-English medical consultation and hospitalization reports authored by physicians. The study included reports for patients with suspected genetic disorders, and non-opposition consent for the use of their clinical data was collected after they were informed about the study by physicians. The first edited or available reports (consultation or hospitalization) in the patient’s electronic health record were included, and their first and last names were pseudonymized before inclusion. We excluded patients who did not provide consent or did not have a consultation or hospitalization report. The choice to enroll 50 patients was informed by precedent in similar software evaluation studies [17, 18]. The institutional review board of University Hospital Center of Montpellier approved the study (IRB-MTP_2023_04_202301351, 14 April 2023).

A physician reviewed 50 French medical reports and initiated a multi-step analysis. First, the proband names were de-identified to ensure privacy. Then, medical reports underwent various processes within the framework, including abbreviation expansion, translation correction, and PHI de-identification according to the Health Insurance Portability and Accountability Act privacy rules. A physician expert compared the method’s outputs with their own independent evaluation. Performance with German and Spanish reports was not evaluated. The evaluation involved assessing the quantity and accuracy of de-identification, translation, and summarization using HPO terms while systematically documenting any errors encountered, specifying their severity, and providing explanations for their occurrence.

We assess the de-identification recall by the method versus the number de-identified by the physician and precision recall metrics of de-identification and symptom summarization in HPO format compared to those for the physician. Errors in the de-identification process were classified as major (e.g. including identifiable information) or moderate (e.g. indirectly identifying PHI), and errors in summarized information were classified as major (e.g. missing the diagnosis) or moderate (e.g. incorrect summarized information). These three levels of errors assessing the performance risk system, inspired by quality assessment ISO 31000:2018, are detailed in Supplementary Table S2.

We considered a missed PHI de-identification as a false negative (FN) and excess de-identification of non-PHI information as a false positive (FP). As well, for the summarization, we considered excess HPO terms as an FP and missed HPO terms as an FN. True positives (TPs) and true negatives (TNs) were de-identification and summarization for which the assessing physician corroborated the tool results.

## Results

### Dataset analysis

**Figure 3.**
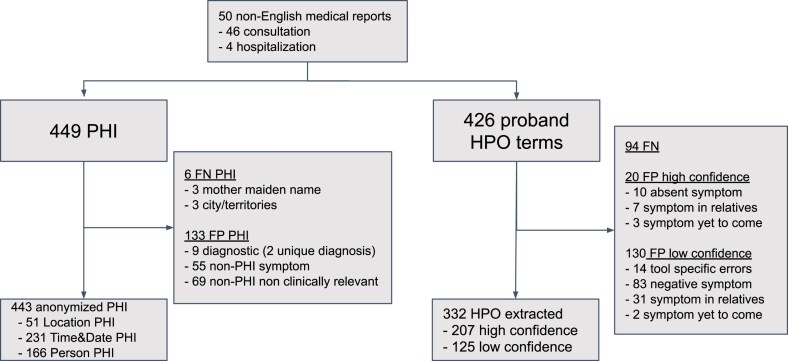
Overview of the analysis. Protected health information de-identification and summarization in Human Phenotype Ontology (HPO) symptom terms from non-English medical reports. PHI, protected health information; FP, false positive; FN, false negative.

From March to April 2023, we consecutively assessed 46 consultation reports and 4 hospitalization reports written between 2019 and 2023 by 8 different physicians. The reports concerned 50 patients, with median age 6 years old, and 12 different consultation reasons, including neurodevelopmental disorders (54%), congenital disorders, fetal pathology, and oncology. The patients included 28 males, 21 females, and one fetus of undetermined sex. The reports contained a median of 478 words, 3180 characters, 3 abbreviations, 15 PHI, and 7 HPO terms. The distribution of the PHI and HPO terms is in Fig. 3. The cohort characteristics are in Supplementary Table S3.

### De-identification performance

ClinFly de-identified 443 of the 449 existing PHI terms, achieving a de-identification recall of 99%; 51 de-identification cases involved the cities/territories dictionary, 231 the date and time detection module, and 166 the person detection module of the software. For the six remaining errors, three were moderate, missing location information (i.e. “Paris”, “Nîmes”), and three others were major, missing the mother’s maiden name of the proband.

ClinFly over-anonymized 133 of 495 non-PHI terms, with the overall de-identification precision of 77% or a de-identification precision of 87% if including only moderate and major errors.

The method de-identified nine non-PHI diagnoses or consultation reasons in excess (i.e. “onychodysplasia,” “Iso Kikuchi”) in the same medical report, 55 non-PHI clinical information terms (symptoms), and 69 non-PHI non-clinically relevant words (“this,” “by,” “morning,” etc.), which were classified as major, moderate, and minor errors, respectively.

### Assessing medical report summaries using the HPO format

ClinFly summarized medical reports with 49% summarization recall and 227 high-confidence HPO terms: 20 were FPs and the summarization precision was 92%. Inaccuracies in the high-confidence HPO terms were primarily due to translation or summarization issues (10 of 20, classified as “major errors”), incorrect interpretation of family member information (7 of 20, “moderate errors”), and premature identification of symptoms yet to manifest, as mentioned in the descriptive paragraphs of the syndrome (3 of 20, not classified as errors). In cases of low-confidence summarizations, 125 of 255 suggested HPO terms were accurate, yielding the summarization precision of 49%. The inaccuracies within the 130 low-confidence HPO terms were attributed to the use of negative phrasing (83 of 130), references to family members (31 of 130), issues with translation or summarization (14 of 130), and premature mention of symptoms not yet presented (2 of 130). Taking into account both high- and low-confidence HPO terms, the method effectively detected 332 of the 426 HPO terms, for a mean of 6.6 HPO terms per report and an overall summarization recall rate of 78%. Additionally, the average F1-scores for high-confidence automated summarization and overall automated summarization were 0.78 and 0.73, respectively. Additional results are available in Fig. 3.

## Discussion

ClinFly addresses key challenges in precision medicine by enabling secure, on-premise translation, de-identification, and HPO-based summarization of medical reports. Our findings show that this integrated pipeline achieves high de-identification accuracy and reliable phenotypic structuring, supporting data-sharing critical for rare disease diagnostics.

To our knowledge, there is no other existing all-in-one framework allowing physicians to translate, de-identify, and summarize medical reports in HPO format. We assessed the potential errors associated with this approach for translating, de-identifying, and summarizing medical reports and compared its performance to that of a physician. The method achieved a 99% recall and 77% precision in de-identifying PHI information, markedly assisting physicians to ensure substantial patient privacy protection for medical report sharing. When summarizing medical reports in HPO format, the software reached the precision of 92%, thus underscoring its accuracy in summarizing symptoms when HPO terms are identified with high confidence. Although human verification may still be necessary to ensure the completeness of the data, particularly for the HPO terms identified with low confidence, the overall effectiveness of this method is notable. Overall, it successfully summarized 332 of the 426 HPO terms, for a mean of 6.6 HPO terms per report. This performance is comparable to the average number of symptoms manually entered by physicians in PhenoDB and the MatchMaker Exchange initiative [19].

Despite thorough efforts, this approach still has its limitations. Flagging terms as “low-confidence” is provisional; a future development priority is to integrate more sophisticated negation detection algorithms to specifically identify and label these terms as “absent” rather than just “low-confidence.” To address this technical limitation, future work could explore benchmarking and incorporating alternative approaches for translation [20], de-identification, and HPO extraction [18, 21]. To meet the broader need for structured health data, this work would benefit from incorporating additional ontologies, such as SNOMED [22]. Currently, the method processes German and Spanish medical reports, but it lacks manual curation improvements for these languages.

In our evaluation study, the primary limitation was that the analysis focused on a single non-English language, French. In addition, the evaluation relied on one expert; future studies should use consensus from multiple clinicians to address inter-rater variability. Additional limitations include the presence of FPs and FNs in both the de-identification and summarization processes. A few instances of PHI, particularly location-related information, were not fully de-identified, thus potentially compromising the anonymity. In addition, the dataset method could be expanded since it does not currently handle specific names of classifications, surgical techniques, and study names, which often leads to over-de-identification and clinical information loss. However, the overall evaluation study gave only a few major errors of de-identification, and enhancements in hybrid processing may resolve these issues.

Regarding summarization in HPO format, FPs could result in incorrect diagnosis prioritization, and FNs could lead to missed prioritization of diagnoses in clinical genome sequencing analysis. Handling multiple patients in a single report remains challenging—especially in genetics, where family histories are common. This likely explains why 35% of summarization errors in our study involved overreporting family member symptoms.

Advances in machine learning, particularly large language models (LLMs), hold significant promise for improving translation, de-identification, and summarization processes. Comparing ClinFly to newer, locally deployed LLMs will be a critical next step to benchmark and advance its performance. As these technologies evolve to align with medical data privacy regulations and resource availability, they are expected to significantly propel the clinical advantages of AI for data sharing and computational phenotype analysis in precision medicine. Despite advancements in LLM, these approaches are currently inadequate for handling extensive health datasets in command-line systems for large-scale data analysis due to privacy concerns, computational limitations, and cost issues. We propose a solution that can be integrated into existing healthcare systems, mitigating the risk of hallucinations, as our errors are explainable.

Overall, this study highlights AI’s potential in facilitating data-sharing for genetic disease research through PHI de-identification and HPO format summarization. AI could help overcome manual entries of clinical data, thus facilitating the exploitation of the “genotype-first” approach in medical genomics to identify new disease genes, expand the clinical spectrum and retro-phenotype the patient’s condition, and confirm the diagnosis with a variant of interest.

## Supplementary Material

lqaf145_Supplemental_File

## Data Availability

This method is open-source and can be installed for offline usage on a local machine by following instructions at https://github.com/kyauy/ClinFly (https://doi.org/10.5281/zenodo.17159057) or is accessible directly online via the command line for large-scale analyses and has a graphical interface for users at https://clinfly.project.erios.ai/. This method currently manages French, German, and Spanish languages. A translated and de-identified report can be downloaded as a text file output. The list of symptoms can be downloaded in CSV or PhenoTips JSON format, compatible with most electronic health record entries, especially in genome sequencing platforms.
