# Supplementary material for: ClinFly: an all-in-one method to translate, de-identify, and summarize medical reports in HPO format
Source: NAR Genom Bioinform. 2025 Nov 3;7(4):lqaf145. doi: 10.1093/nargab/lqaf145 (PMC12582024; doi:10.1093/nargab/lqaf145)
Supplement: lqaf145_Supplemental_File [file lqaf145_supplemental_file.pdf]

Supplementary material and methods

Methods S1. Additional information on medical letter processing using ClinFly

To ensure accurate extraction of clinical concepts, we implemented language-specific customizations to adapt to non-English grammar and phrasing, and filtering of abbreviations to avoid misinterpretation. Concerning abbreviation, we have listed 146 frequent French country-specific abbreviations, usually stand for pathologies, medical related structures, technologies and analysis, collected from Geneva University Hospitals' glossary (http://abreviationsmedicales.ch/), as well as the collaborative Wikipedia page "Liste d'abréviations en médecine" (https://fr.wikipedia.org/wiki/Liste_d%27abr%C3%A9viations_en_m%C3%A9decine), and the Pays de la Loire Regional Health Agency (ARS) abbreviation list (https://www.pays-de-la-loire.ars.sante.fr/system/files/2018-06/Aide%20-%20Acronymes.pdf), to write them out in full in French and translate them into English. Five among those cannot be expanded because of their ambivalent meaning (*e.g.* TCA, CMT, RCP). To ensure an accurate translation of the clinical information, a correction dictionary composed of 4646 terms was created from the French translation of Human Phenotype Ontology (accessible in PhenoTips website https://nexus.phenotips.org/nexus/content/repositories/releases/org/phenotips/vocabulary-hpo-translation-french/1.4-rc-4/, October 2018 release) and manually reviewed. 163 translations have been removed because of misleading translation without alternative, 54 have been replaced. This human-reviewed system is detailed in Table S1.

The inclusions were performed consecutively, resulting in the least possible bias.

To ensure accuracy, we implemented specific measures such as strict separation of abbreviations with spaces during expansion. Furthermore, we replaced the term "associated" with "with" to avoid potential errors resulting from considering it to indicate a disease name. Additionally, punctuation-related issues prompted us to replace abbreviations such as "M.," "Dr.," and "Pr." with appropriate alternatives.

To ensure the preservation of clinical information, we implemented specific exclusions for the de-identification of dates and times: this involved retaining temporal words (e.g., years, months, noon, hours) and slash-related information (e.g., blood pressure 140/80, Apgar score 7/7/8/10) that do not represent specific dates (e.g., 10/12/1994). Considering the low likelihood of license plate and number mentions in consultation letters, we deactivated their detection to minimize the risk of misidentifying relevant information (e.g., genes, height).

Furthermore, to maintain anonymity, the proband's first and last names were pseudonymized as "CAS" and "INDEX," respectively, thus ensuring a clear distinction from other anonymous individuals.

ClinFly uses a clause-by-clause analysis approach, employing punctuation marks such as commas and periods to effectively manage false-positive summarization, particularly from negative sentences and information related to the patient's family members.

Our systematic summarization method focuses on the first sentence of the consultation letter, which commonly contains the primary reason for the patient's visit, while also considering any relevant clinical details pertaining to their relatives.

Notably, an upgraded version of ClinPhen was developed to address errors related to HPO_ID synonyms.

Methods S2. Technical report

Implementation details:

ClinFly is an automated framework for precision medicine in genetic diseases, implemented primarily in Python 3.8 or higher, available open-source on GitHub (https://github.com/kyauy/ClinFly). A graphical summary of the framework is provided in figure 1.

It uses several key libraries, pre-trained models and frameworks:

Stanza: For natural language processing tasks, particularly sentence tokenization (https://stanfordnlp.github.io/stanza/).

Transformers: pre-trained MarianMT model for machine translation (https://marian-nmt.github.io/).

Presidio: Microsoft's tool for Personal Health Information (PHI) de-identification (https://microsoft.github.io/presidio/).

Streamlit: For the web-based graphical user interface (https://streamlit.io/).

ClinPhen: For extracting Human Phenotype Ontology (HPO) terms (http://bejerano.stanford.edu/clinphen/).

The ClinFly pipeline integrates several steps:

Input: Clinical reports in various languages (currently optimized for French to English).

Pre-processing steps: Includes handling abbreviations and reformatting text for better translation.

De-identification: Uses Presidio Analyzer and Anonymizer to detect and remove personal information.

Translation: Employs the MarianMT pre-trained model to translate non-English reports to English.

Post-processing step: Includes adding biometric information and reformatting the translated text.

HPO Term Extraction: Utilizes ClinPhen to summarize clinical information using standardized HPO terms.

Output: Produces de-identified translated reports and HPO-formatted summaries.

Focus on Microsoft Presidio for PHI De-identification:

Input Details: Raw clinical reports containing personal health information, including names, dates, and locations.

Output Generated: De-identified text with sensitive information removed or replaced. The code uses custom configurations to handle additional cities and specific medical terms we provided. The output is then used in subsequent translation and HPO extraction steps.

Computational Resources:

Requirements included in requirements.txt or pyproject.toml file in Github repository (https://github.com/kyauy/ClinFly)

Python >= 3.8

Libraries: stanza, transformers, presidio, streamlit, pandas, unidecode

Pre-trained models: MarianMT translation model, Spacy English language model (en_core_web_lg), presidio_anonymizer and presidio_analyzer

ClinFly is usable on laptops and personal computers. The online ClinFly virtual machine uses 16GB ram and 2 CPU.

Benchmark of translation and summarization in HPO format tools :

We benchmarked two machine-translation engines— commercial solution DeepL (https://www.deepl.com/fr/translator, November 2022 version), and open-source and on-premise model MarianTranslator—and three phenotype extractors—ClinPhen, txt2HPO

(https://github.com/GeneDx/txt2hpo), and Doc2HPO (Liu et al. 2019). Translations of the same anonymised clinical report produced by each engine were processed by every extractor. All HPO terms were manually curated to verify their presence in the source text.

A exclusive random sample of ten medical reports was analyzed for translation and summarization. W report the non-inferiority of MarianTranslator relative to DeepL to translate and identify the optimal extractor. MarianTranslator with custom dictionaries retrieved 15% more HPO terms than DeepL with an identical 8% false-positive rate, reducible to 2% through targeted dictionary updates. Regarding extractor performance, ClinPhen and txt2HPO showed similar yields (respectively 105 and 109 HPO terms retrieved) and false positives, most correctable via minor edits. Given its performance and integration ease, the MarianTranslator–ClinPhen pipeline was selected, with a final 8.9% false-negative rate, supporting its suitability in large-scale clinical settings.

Supplementary Tables

Table S1. Curated resources for hybrid method

Collection of human-reviewed dictionaries included in the process for managing translation and de-identification issues. The table highlights the content count, required sources, and provides an example of the dictionaries' functionality.

Steps	Count	Sources	Example
**Translation issue**<br>Getting full and accurate informations			
Abbreviations	146	Geneva University Hospitals' abbreviation glossary<br>Pays de la Loire Regional Health Agency (ARS) abbreviation list<br>Wikipedia page "Liste d'abréviations en médecine	TSA → autisme
Translation errors	4646	Human Phenotype Ontology	callous body → corpus callosum
**De-identificaiton issue**<br>Not to miss PHI and avoid over-anonymization of clinical informations			
Proper name: syndrome	1263	OMIM	Optiz → Optiz
Proper name: symptom	1735	Human Phenotype Ontology	Gowers → Gowers
Drug	4939	ANSM lists of officinal medication	Abilify → Abilify
Gene	109804	HGNC approved gene dataset	*MECP2* → *MECP2*
French territories	34981	Insee french official geographic code	Pau → [FRENCH_CITY]

Table S2. Assessing type of errors of de-identification and summarization of medical reports in three-levels of severity

Severity	De-identification missed	Excess of de-identification	Summarization missed	Excess of summarization
**Minor**		Loss of sentence fluidity		Future potential symptoms
**Moderate**	PHI	Loss of a symptom	Loss of a symptom	Family member symptom
**Major**	name / mother's maiden name	Loss of the consultation reason or the diagnostic	Loss of the consultation reason or the diagnostic	Negative sentences or translation & summarization issue

Table S3. Study cohort

Characteristics of the medical reports used for analysis.

Cohort	n [min_max]
Number of physicians	8
Number of medical reports	50
Number of consultation reasons	12
Median age in report	6 [0-50]
**Metrics per report in median**	**n [min_max]**
Words	478 [229-1728]
Characters	3180 [1571-11405]
Abbreviations	3 [0-14]
PHI	15 [5-70]
HPO terms	7 [2-26]
